# Vitrectomy Combined With Gas Tamponade for the Treatment of Morning Glory Syndrome With Rhegmatogenous Retinal Detachment: A Case Report

**DOI:** 10.7759/cureus.78550

**Published:** 2025-02-05

**Authors:** Wen-Jian Xin, Tong-tong Niu

**Affiliations:** 1 Department of Ophthalmology, Xinjiang 474 Hospital, Xinjiang, CHN

**Keywords:** c3f8 gas, morning glory syndrome, rhegmatogenous retinal detachment, treatment, vitrectomy

## Abstract

We report a case of a 26-year-old male patient who was diagnosed with morning glory syndrome (MGS) in his right eye through a series of specialist examinations. At the time of consultation, the patient had already experienced retinal detachment in his right eye. The fundus of the right eye showed a 360° grayish-white elevation of the entire retina, with an enlarged optic disc approximately 4-5 times the normal size, featuring a central funnel-like deep depression. The surface of the optic disc was covered with grayish-white glial tissue, and elevated ridges with pigmentation were visible around the disc. Several straight vessels radiated from the edge of the optic disc, making it difficult to distinguish between arteries and veins. A comprehensive preoperative fundus examination failed to uncover any conclusive retinal tears. We performed a 25-gauge vitrectomy through the pars plana, and during the surgery, a small retinal tear was found at the nasal edge of the optic disc. After air-fluid exchange, laser photocoagulation of the retina was performed around the edge of the optic disc, followed by injection of 14% C3F8 gas. One month postoperatively, the gas was completely absorbed, and after a six-month follow-up, the retina was well-repositioned.

## Introduction

Morning glory syndrome (MGS) is a rare congenital anomaly of the optic disc, characterized by optic nerve defects, unique retinal vascular abnormalities, and pigmentary changes around the optic disc. Like ocular coloboma, MGS is thought to be associated with mutations in the PAX6 gene. Retinal detachment is often secondary to MGS [1-2]. Studies have reported that 26-38% of patients with MGS have retinal detachment [3]. For retinal detachment associated with MGS, the retinal breaks are mostly located near the optic disc. However, due to the small size of the breaks and the similar appearance of the optic disc in MGS, the breaks are often difficult to detect preoperatively [4-5]. The surgical approach is vitrectomy through the pars plana, and some scholars use vitrectomy combined with silicone oil injection to flatten the retina, followed by silicone oil removal after retinal flattening [5]. The patient in this case is a young male and his right eye was diagnosed with MGS, and no definite retinal tear was detected prior to the surgery. We performed vitrectomy and, considering the complications associated with silicone oil tamponade, such as complicated cataract and secondary glaucoma, as well as the patient's young age and aversion to silicone oil, after vitrectomy, we injected the inert gas perfluoropropane (C3F8) at a concentration of 14%. One month postoperatively, the gas was absorbed completely, and the retina was well-repositioned without ocular complications or systemic adverse events.

## Case presentation

Case presentation

This was a 26-year-old male patient with a history of right eye deviation for more than 10 years, for which he had not sought medical attention. He planned to improve his appearance before marriage and therefore consulted the ophthalmology center of Xinjiang No. 474 Hospital.

Ocular examination

**Figure 1:**
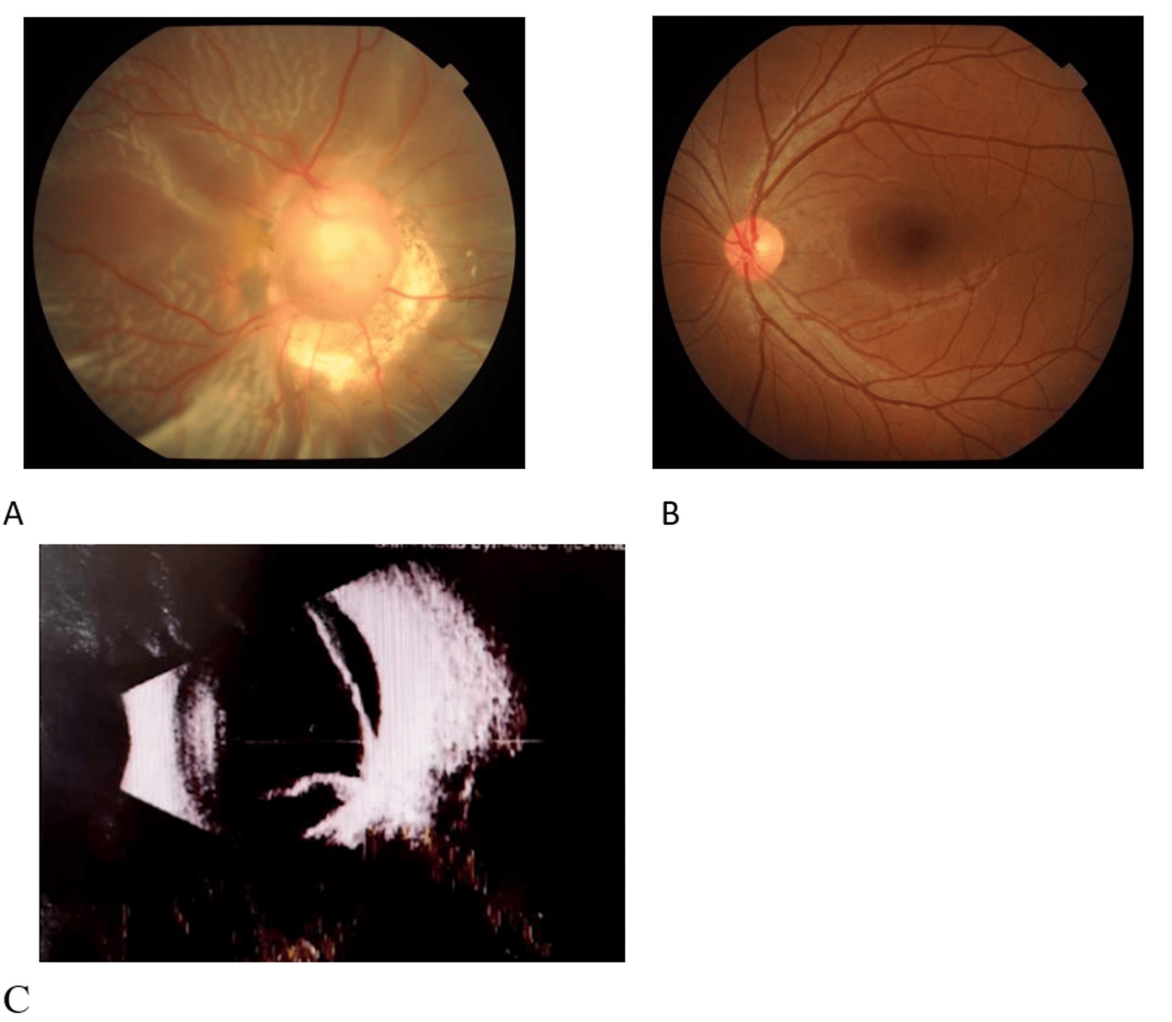
Preoperative ocular examination (A) Preoperative: Fundus Photography: The optic disc of the right eye appears large with a deep depression, covered by a grayish-white glial tissue on its surface, obscuring the vascular pattern within the optic disc. Several straight blood vessels emanate radially from the disc margin, making it difficult to distinguish arteries from veins. There is total retinal detachment. (B) The fundus of the left eye shows no significant abnormalities. (C) B-scan Ultrasonography of the Right Eye: Total retinal detachment is present, exhibiting a "funnel-shaped" alteration.

OD: HM (uncorrected visual acuity), OS: 20/20 (uncorrected visual acuity), with no improvement after correction. Intraocular pressure: OD 15 mmHg, OS 16 mmHg (1 mmHg = 0.133 Kpa). Both eyes had normal ocular motility, with the right eye deviated outward by 30° and the left eye in normal position. On slit-lamp examination, the conjunctiva of both eyes exhibited no hyperemia. The corneas were transparent, the anterior chambers were clear, and the pupils were round. The pupillary light reflex was sluggish in the right eye, whereas it was sensitive in the left eye. Fundus examination of the right eye revealed a "funnel-shaped" retinal detachment extending from an enlarged and deeply depressed optic disc, approximately 4-5 times the normal size. The surface of the optic disc was covered with grayish-white glial tissue, with elevated ridges and pigmentation around the disc. Several straight vessels radiated from the edge of the optic disc, making it difficult to distinguish between arteries and veins (Figure 1A). The left eye showed no obvious abnormalities (Figure 1B).

B-scan ultrasonography of the right eye showed a "funnel-shaped" retinal detachment. The patient had no history of underlying diseases, ocular surgery, or ocular trauma. Previous genetic testing revealed a PAX6 gene mutation.

**Figure 2:**
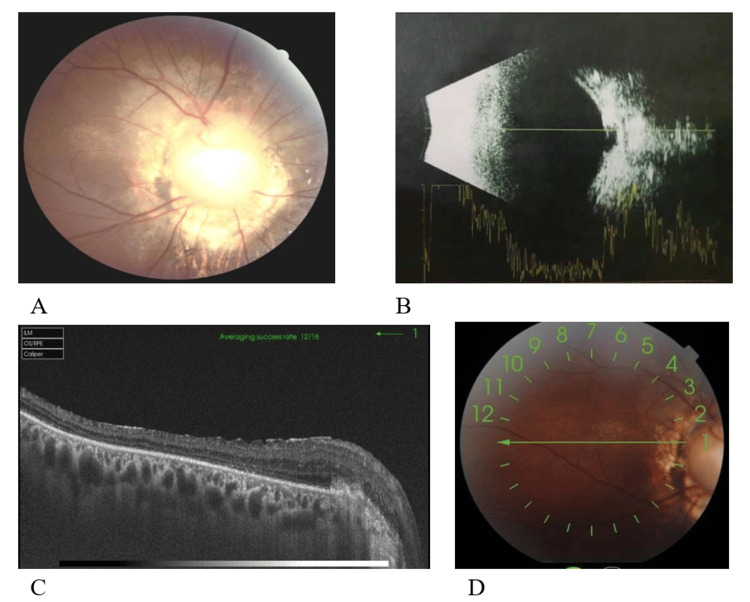
Postoperative ocular examination one month after surgery A: One month post-surgery, fundus photography of the right eye shows the retina has been reattached with laser spots visible around the optic disc. Partial macular structure is preserved, and the best-corrected visual acuity is 2/100. B: B-scan Ultrasonography of the right eye post-surgery: The retina has been reattached, and a depressive change of the optic disc is visible. C & D: OCT of the macular area (circular scan) of the right eye one month post-surgery: The macular area exhibits developmental abnormalities, with no central foveal morphology visible. The retina is atrophic and thinned.

**Figure 3:**
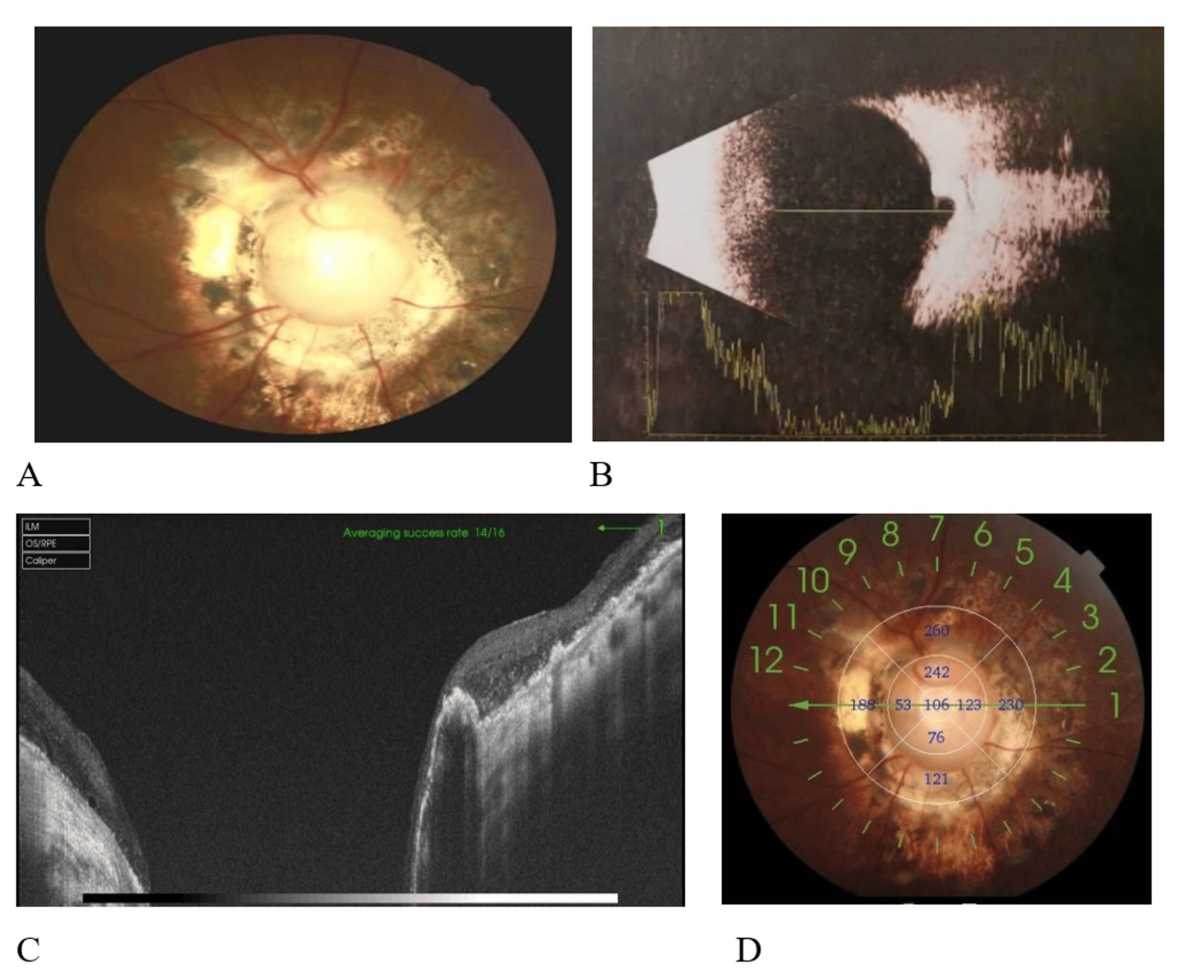
Postoperative ocular examination six months after surgery (A) At six months post-surgery, fundus photography of the right eye revealed normal retinal morphology. Pigment hyperplasia and atrophic thinning of the retina around the optic disc were visible, with a best corrected visual acuity of 2/100. (B) B-mode ultrasound examination of the right eye demonstrated retinal reattachment, with visible concave changes in the optic disc. (C and D) Optical coherence tomography (OCT) scans of the optic disc showed retinal reattachment.

We performed a 25-gauge minimally invasive vitrectomy through the pars plana, with meticulous removal of the vitreous and careful dissection of the peripheral vitreous to prevent iatrogenic retinal breaks. After staining the vitreous with triamcinolone acetonide, the residual vitreous cortex was slowly removed. Intraoperatively, triamcinolone acetonide suspension particles deposited on the depressed surface of the optic disc. A flute needle was used for repeated aspiration and blowing to explore for retinal breaks and strip away the glial fibrous tissue on the surface of the optic disc (which was critical for surgical success). A small retinal tear was found at the nasal edge of the optic disc. During air-fluid exchange, viscous fluid was seen emanating from the break. Laser photocoagulation was performed 360° around the optic disc, followed by injection of 14% C3F8 gas. The patient was instructed to maintain a prone position for three weeks. One month postoperatively, the inert gas was completely absorbed (Figure 2). During the six-month postoperative follow-up (Figure 3), the retina was well reattached, the intraocular pressure was normal, and the best-corrected visual acuity was 20/400. No ocular complications or systemic adverse reactions were observed.

## Discussion

In 1970, Kindler first described MGS, named for its resemblance to a "fully bloomed morning glory" on the fundus. The pathogenesis of MGS is unknown, but it may be caused by the failure of the uppermost part of the embryonic fissure to close, resulting in the protrusion of the optic disc and surrounding tissues backward. Alternatively, it may be associated with abnormal glial development in the central fovea of the optic disc, and it mostly affects one eye [6]. MGS typically occurs in children and adolescents with no gender preference. It is often present at birth, but due to infants' inability to cooperate with examinations and parental negligence, it is often not detected promptly. It is usually diagnosed when strabismus, low vision, and nystagmus appear. Typical MGS patients have an abnormally enlarged optic disc with a pink edge shaped like a funnel. There is a dense, structurally insignificant white area in the center resembling the flower's pistil, obscuring the deeper blood vessels. Surrounding the optic disc is a ring of grayish-white or gray-black protrusions, like a wreath, accompanied by scattered pigment spots. Sometimes, there is also retinal-choroidal atrophy. Additionally, about 20 to 30 blood vessels radiate outward from the edge of the optic disc. These features collectively constitute the typical manifestations of MGS [1, 6]. Radiologically, MGS also exhibits specific features. Magnetic resonance imaging (MRI) results show that most eyes with MGS have similar characteristics, with the optic disc showing inward depression and a funnel-shaped or cystic dilation of the optic papilla, communicating with the vitreous body and sharing the same fluid signal [7]. B-scan ultrasonography of the eyes further reveals these details: the optic disc and its surrounding tissues are concave and enlarged toward the posterior part of the globe, making the posterior vitreous cavity look like an inverted "bottle neck" with a clearly identifiable edge and a defined boundary at the bottom of the depression. Inside this depression, irregular and weak echoes can be seen [8].

Apart from ocular abnormalities, MGS often involves other systemic systems such as the nervous system, cerebrovascular system, and endocrine system. The most common neurological abnormality is basilar encephalocele [7-9]. Lenhart et al. found that the incidence of varying degrees of cerebrovascular abnormalities in patients with MGS is as high as 45%, including moyamoya disease, hypoplasia of the corpus callosum, and hypopituitarism [7]. Moyamoya disease is caused by progressive stenosis of the intracranial internal carotid artery, leading to compensatory collateral circulation in the basilar arteries. During angiography, these collateral vessels produce a pathological appearance known as the "moyamoya" pattern. Basilar encephalocele is often associated with midface abnormalities, ocular abnormalities, brain abnormalities, cerebrospinal fluid rhinorrhea, optic chiasm syndrome, and hypothalamic-pituitary dysfunction [9-10]. Komiyama et al. reported a 29-year-old male with basilar encephalocele (transsphenoidal type), MGS, panhypopituitarism, and moyamoya disease [11]. Due to stable ocular signs and no cerebrospinal fluid leakage or respiratory distress, the patient received conservative hormone therapy without surgical intervention. Solomou et al. [12] reported a 2.5-year-old Greek girl previously diagnosed with MGS who presented with right-hand paralysis accompanied by focal epileptic spasms, followed by brief absences. Magnetic resonance angiography scans showed an abnormal origin of the anterior cerebral artery from the internal carotid artery, accompanied by vascular abnormalities consistent with moyamoya disease, suggesting a potential pathological association between congenital optic disc abnormalities and moyamoya disease. Researchers using deep learning models with real-world data have identified fundus retinal photographs as potential biomarkers for moyamoya disease screening and staging, and the disease stage can be classified through deep learning algorithms [13]. We conducted a cerebral MRA examination on this patient and found no abnormalities.

In clinical practice, differentiating MGS from the following diseases is of great significance: (1) Congenital Optic Disc Coloboma: The optic disc manifests a white depression of the inferior optic nerve, which might extend inferiorly to involve the choroid and retina. The superior margin of the optic nerve remains usually normal, and both the size of the optic disc and its associated blood vessels generally maintain normal states. (2) High Myopia Complicated by Posterior Scleral Staphyloma: Patients typically suffer from high myopia, characterized by a blurred optic disc border and tortuous blood vessels. Multiple choroidal atrophy foci can be observed in the retina, which gradually thins, exposing the coarse choroidal vessels. The branching patterns of the retinal blood vessels exhibit no abnormalities. (3) Congenital Megalopapilla: The optic papilla is remarkably large, approximately twice the size of a normal one, with a normal physiological cup. A narrow, circular arcuate patch encircles the optic papilla, and the macula remains in a normal condition. The retina and blood vessels are relatively more delicate. (4) Choroidal Osteoma: In the vicinity of the optic disc, a yellowish-white mass with an uneven surface exists. Fluorescein angiography demonstrates early-stage granular and plaque-like hyperfluorescence, which persists throughout the late stage. (5) Primary Optic Disc Drusen: The optic disc appears as a mulberry-like aggregation of drusen. These drusen are translucent, vary in size, and may protrude into the vitreous cavity.

Currently, one of the primary causes of rapid vision decline in patients with MGS is retinal detachment. However, the mechanism underlying secondary retinal detachment in MGS is still unclear. It is speculated that small pits formed by abnormal connective tissue on the optic disc allow cerebrospinal fluid from the subarachnoid space to flow down and cause retinal detachment. Another mechanism may involve the hyperplasia and traction of fibrous tissue on the surface of the abnormally developed optic disc. This traction force may be one of the causes of retinal holes. The vitreous tracts the thin atrophic membrane around the optic disc, leading to the formation of secondary retinal holes. Fluid from the vitreous cavity enters the subneuroepithelial layer through slit-like retinal holes located at the edge of the optic disc protrusion, causing retinal detachment [14]. A considerable proportion of pediatric patients with MGS have retinal detachment, and the holes are often difficult to detect. In recent years, with the application of optical coherence tomography (OCT) equipment, it has been found that tiny holes at the edge of the optic disc are the main cause of retinal detachment. Due to the rare incidence of MGS, there are currently not many reports on surgical interventions. Yu et al. [15] reported on a 3-year-old girl with MGS who had no systemic abnormalities after a comprehensive examination, but no mention was made of subsequent treatment. Zheng et al. [16] reported on a 5-year-old child with MGS combined with persistent hyperplastic primary vitreous (PHPV). The child's family refused surgery after being informed that the surgery would not improve vision. Etheridge et al. [17] performed vitrectomy combined with silicone oil injection on a 19-year-old patient with MGS and retinal detachment. Postoperative follow-up showed that the patient's retina was flat, and there was no decrease in vision. Zou et al. [18] treated 24 eyes of 22 consecutive patients with adolescent MGS with prophylactic near-optic nerve head laser photocoagulation. The study suggested that prophylactic laser photocoagulation for near-optic nerve head in pediatric patients with MGS showed relatively stable initial anatomical and visual outcomes during short-term follow-up. However, further long-term clinical observation is needed to assess its efficacy and safety.

Retinal detachment is unlikely to occur without vitreous or optic disc abnormal glial tissue causing retinal traction. Therefore, we believe that surgery is required to relieve retinal traction and close the holes. Preoperatively, we performed repeated OCT scans on this patient. Due to the "funnel-shaped" detachment of the entire retina, retinal folds and distortions, along with a deep optic disc depression, the holes were not found. However, intraoperatively, using repeated blowing and aspiration with a flute needle, we finally found a small hole on the nasal side of the optic disc edge, which corroborates reports in the literature that holes are mostly located at the optic disc edge [7]. During the entire surgical procedure for this patient, despite the challenges posed by complete retinal flotation and swinging, we did not use heavy water to assist in fixing the retina to avoid heavy water droplets entering the subarachnoid space or even the ventricular cavity. Considering the toxicity of silicone oil and the potential for cerebrovascular accidents, we chose to fill the eye with 14% C3F8 gas at the end of the surgery. Postoperative follow-up for six months showed good retinal repositioning, with no ocular or systemic adverse events. The treatment of retinal detachment was achieved with a single surgery. Although this is the only successfully treated case, we still recommend attempting it in clinical practice. It is worth emphasizing that the key to surgical success lies in completely removing the vitreous cortex on the retinal surface and the glial tissue on the optic disc surface, effectively relieving vitreous traction, and closing the holes at the optic disc edge.

## Conclusions

In conclusion, MGS is a congenital optic disc anomaly with a relatively low incidence, and it is often complicated by secondary retinal detachment. For such patients, in most cases, vitrectomy combined with silicone oil injection is adopted. The case we reported this time indicates that for patients with MGS complicated by retinal detachment, vitrectomy combined with C3F8 injection is also safe and reliable. This not only reduces postoperative complications but also alleviates the psychological and financial burden on patients caused by the need for a second-stage silicone oil removal procedure, and it can be applied in clinical practice.
